# Human Nucleoporins Promote HIV-1 Docking at the Nuclear Pore, Nuclear Import and Integration

**DOI:** 10.1371/journal.pone.0046037

**Published:** 2012-09-25

**Authors:** Francesca Di Nunzio, Anne Danckaert, Thomas Fricke, Patricio Perez, Juliette Fernandez, Emmanuelle Perret, Pascal Roux, Spencer Shorte, Pierre Charneau, Felipe Diaz-Griffero, Nathalie J. Arhel

**Affiliations:** 1 Molecular Virology and Vaccinology Unit, CNRS URA 3015, Department of Virology, Institut Pasteur, Paris, France; 2 Imagopole, Institut Pasteur, Paris, France; 3 Department of Microbiology and Immunology, Albert Einstein College of Medicine, Bronx, New York, United States of America; University of South Carolina School of Medicine, United States of America

## Abstract

The nuclear pore complex (NPC) mediates nucleo-cytoplasmic transport of macromolecules and is an obligatory point of passage and functional bottleneck in the replication of some viruses. The Human Immunodeficiency Virus (HIV) has evolved the required mechanisms for active nuclear import of its genome through the NPC. However the mechanisms by which the NPC allows or even assists HIV translocation are still unknown. We investigated the involvement of four key nucleoporins in HIV-1 docking, translocation, and integration: Nup358/RanBP2, Nup214/CAN, Nup98 and Nup153. Although all induce defects in infectivity when depleted, only Nup153 actually showed any evidence of participating in HIV-1 translocation through the nuclear pore. We show that Nup358/RanBP2 mediates docking of HIV-1 cores on NPC cytoplasmic filaments by interacting with the cores and that the C-terminus of Nup358/RanBP2 comprising a cyclophilin-homology domain contributes to binding. We also show that Nup214/CAN and Nup98 play no role in HIV-1 nuclear import *per se*: Nup214/CAN plays an indirect role in infectivity read-outs through its effect on mRNA export, while the reduction of expression of Nup98 shows a slight reduction in proviral integration. Our work shows the involvement of nucleoporins in diverse and functionally separable steps of HIV infection and nuclear import.

## Introduction

The nuclear pore complex (NPC) is a supramolecular protein assembly forming a highly selective channel embedded in the nuclear membrane. It regulates bidirectional nucleo-cytoplasmic transport for a large range of proteins and complexes too large to diffuse freely through the NPC [1], [2], [3]. They are composed of numerous copies of ∼30 different nucleoporins (Nups), which have a well-assigned localisation, function and half-life, and are present as multiples of eight reflecting the highly conserved eight-fold axial symmetry of NPCs [2], [4], [5], [6].

The central substructure of the NPC is composed of transmembrane Nups that anchor the NPC to the nuclear envelope, scaffold Nups (e.g. Nup107/160 complex) that constitute cornerstones during NPC biogenesis, and FG-Nups (e.g. Nup98, Nup358/RanBP2, Nup214/CAN) so-called because they contain extensive repeats of phenylalanine-glycine (FG) domains that form an unstructured mesh at the centre of the channel [6]. Nup358/RanBP2 and Nup214/CAN have been mapped exclusively to the cytoplasmic side of the NPC, where 50–100 nm long flexible cytoplasmic filaments radiate from the NPC into the cytoplasm. Nup358/RanBP2 has been reported to be the major component of the cytoplasmic NPC filaments [7]. Nup98 is a symmetrical nucleoporin, located on both the cytoplasmic and nuclear sides of the NPC [8]. On the nuclear side of the NPC, Nups such as Nup153 and Nup98 associate with the nuclear basket and with the chromatin both in proximity of and away from the NPC [9].

Many viruses depend on access to the nuclear compartment for replication and have evolved unique strategies to translocate into the nucleus [10], [11]. Retroviruses such as Murine Leukaemia Virus (MLV) enter the nucleus during mitotic nuclear membrane disassembly, however other viruses such as herpesviruses and adenoviruses dock their capsids at the NPC and release their genome into the nucleus, while still others (e.g. SV40 and baculovirus) enter in the nucleus with their capsid. The Human Immunodeficiency Virus type 1 (HIV-1), contrary to other orthoretroviruses, has evolved the ability to infect non-dividing cells through active nuclear import of its genome across the intact nuclear membrane through the NPC [12]. Although several viral elements have been proposed to act as determinants of HIV-1 nuclear import, most notably integrase (IN) and the central DNA Flap [13], it is commonly accepted that HIV-1 depends on host cell proteins to achieve translocation. Previous studies have shown the implication of several nucleoporins (Nup62, Nup85, Nup98, Nup107, Nup133, Nup153, Nup160, Nup214/CAN, and Nup358/RanBP2) in HIV-1 nuclear import and/or infectivity [14], [15], [16], [17], [18], [19], [20]. However, the mechanistic implication and individual contribution of these apparently redundant functions remain to be clarified.

Intrigued by the apparent redundancy of Nups potentially assisting HIV-1 translocation through the nuclear pore, and challenged by the lack of mechanistic implications for these Nups, we set out to determine the involvement of four key Nups (Nup358/RanBP2, Nup214/CAN, Nup98 and Nup153) in functionally separable steps of HIV-1 infection and nuclear import. We found that although all four Nups induced defects in HIV-1 infectivity when depleted, only Nup153 actually showed any evidence of participating in HIV-1 translocation through the nuclear pore, probably assisting HIV-1 to exit from the nuclear basket and integrate in the host chromosomal genome possibly in concert with Nup98. Further, we provide evidence that Nup358/RanBP2, identified in a functional genomic screen [17], mediates HIV-1 core docking at the nuclear pore by interacting with capsid. Investigation of the mechanistic role in HIV-1 infection of Nup214/CAN, which was found in another screen [18], revealed that it only plays an indirect role through its effect on mRNA export. Our work sheds light on the participation of key nucleoporins in HIV-1 infection in functionally separable steps of nuclear import.

## Results

### Efficient Knock-down of Human Nucleoporins using Lentiviral Vector-encoded shRNAs

**Figure 1:**
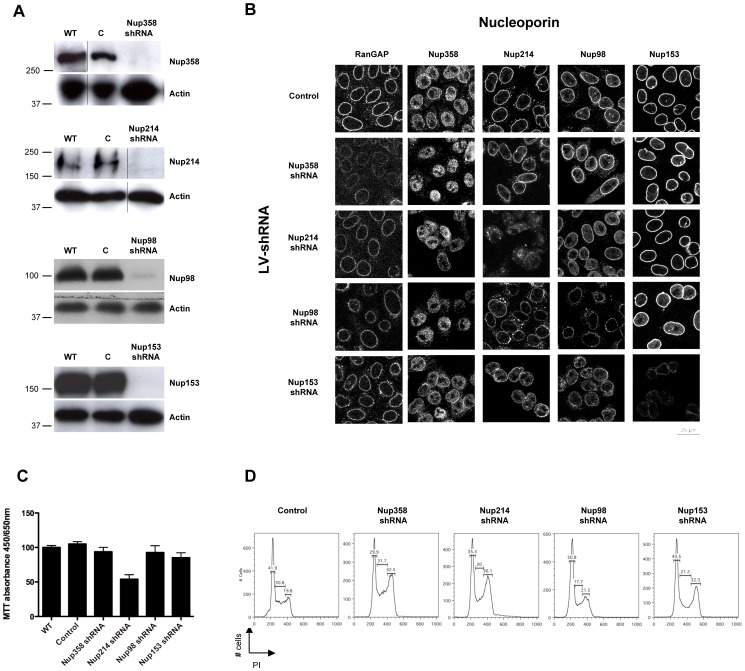
Lentiviral vector-encoded shRNAs achieve efficient knock-down of human nucleoporins and have negligible cytotoxic or cytostatic effects. Hela cells (4×10^6^) were transduced with lentiviral vectors (MOI 50) encoding shRNAs specific for the indicated nucleoporins and used at 2 days p.t for Nup153 shRNA and 5 days p.t for all others. (**A**) Knock-down was assessed by Western blotting using specific antibodies against the targeted nucleoporins in non-transduced (WT), LV-transduced (C) and LV-shRNA transduced cells. ß-actin labelling serves as loading control. Results are representative of at least 3 independent experiments. Numbers to the left of Western blots indicate sizes in KDa. (**B**) Subcellular localisation of nuclear pore components upon nucleoporin knock-down was tested by confocal fluorescence microscopy of LV- (Control) and LV-shRNA transduced cells using specific anti-Nup antibodies. Images were acquired on the same day with the same conditions and are representative of two independent experiments. (**C**) Cell viability was determined by detecting mitochondrial activity in living cells using the MTT assay. Results show the mean of two experiments carried out in triplicates +/− SD. (**D**) Cell cycling was assessed by propidium iodide labelling followed by flow cytometry. x and y ordinates show propidium iodide fluorescence and cell counts, respectively.

We constructed lentiviral vector-based small hairpin RNA (LV-shRNA) by inserting shRNA sequences (targeted against Nup358/RanBP2, Nup214/CAN, Nup98 or Nup153, Table S1) downstream of the H1 promoter in the U3 region of the 3′ LTR of the HIV-1-derived vector TRIP-CMV-eGFP. Efficiency of RNA interference was assessed by Western blotting using specific anti-Nup antibodies in LV-shRNA transduced cells compared to non-transduced cells (WT) and cells transduced with LV alone (C) (**Fig. 1A**). Based on considerations of nucleoporin half-life and stability, as well as cell viability, all knock-down Nup cells were used at 5 days post-transduction (p.t) except for Nup153 knock-down (KD) (2 days p.t), at which time points GFP expression, used to identify transduced cells, was >95%. Contrary to other nucleoporins, Nup153 depletion was deleterious to cell viability after 7 days. Since efficient knock-down was already possible at 2 days post-infection (**Fig. 1A**), we chose to use all Nup153 knock-downs at this point comparing them with the corresponding control. Other nucleoporins required a further 5 days after transduction for efficient knock-down possibly because they have a longer half life.

To investigate the effects of nucleoporin depletion on the expression and distribution of other nucleoporins, we carried out a series of immunolabelling reactions followed by confocal fluorescence microscopy (**Fig. 1B**). These were carried out as a set and in the same conditions to allow comparisons of nucleoporin intensity as well as localisation between different LV-shRNA transduced cells. In control cells, nucleoporin labelling generated a characteristic nuclear rim fluorescent signal. Nup98 was also detected in the nucleus, which is concordant with previous studies showing nuclear localisation of Nup98 [21], [22], [23]. Because of high background of the RanBP2 antibody in immunofluorescent labelling, we also used labelling of RanGAP-1, known to form a complex with Nup358/RanBP2 during interphase and metaphase [24], [25], [26], [27], as an indirect marker for Nup358/RanBP2 localisation. As Nup358 provides a binding site for sumoylated RanGAP at the NPC [24], [25], depletion of Nup358 leads to a concomitant loss of RanGAP from the nuclear pore. As expected, the knock-down of Nup358/RanBP2 led to a strong reduction in RanGAP1 perinuclear labelling (**Fig. 1B**). On the whole, depletion of specific nucleoporins had limited impact on the expression of the other nucleoporins tested and their correct incorporation in NPCs. In some cases, however, some perturbation in nuclear rim labelling could be observed. For instance, Nup98 depletion induced slight perturbations in Nup214 and Nup153 nuclear rim staining, with increased detection of Nup214/CAN in the cytoplasm and of Nup153 in the nucleus (**Fig. 1B**). This may be due to the concomitant depletion of Nup96, since Nup98 and Nup96 are translated as a polyprotein from the same messenger RNA [28] and Nup96 is a component of the Nup107–160 complex. Moreover, Nup214/CAN depletion led to a slight reduction of RanGAP1 at the nuclear envelope, which is concordant with a previous report that Nup214/CAN stabilises Nup358/RanBP2, and that RNA interference of Nup214/CAN reduces Nup358/RanBP2 levels at the nuclear envelope [29].

We next tested the effect of transduction and nucleoporin depletion on cell viability using an MTT colorimetric assay that measures mitochondrial activity in living cells, through the conversion tetrazole into formazan salts by intracellular NAD(P)H-oxidoreductases. We found no notable differences between knock-down and control cells at the time of infection, except for Nup214 KD cells which had 50% reduced viability (**Fig. 1C**). Results discussed subsequently provide some explanation for this cytotoxicity. Furthermore, since some nucleoporins are involved in mitotic progression [5], we tested the effect of nucleoporin knock-down on cell cycling. Flow cytometry profiles following propidium iodide labelling revealed no cell cycle arrest at 4, 5 or 7 days post transduction (p.t.) for any of the transduced cells (**Fig. 1D** and data not shown). A slight increase in G1/S population cells was noted for Nup98 knock-down cells and may be accounted for by the concomitant knock-down of Nup96 since depletion of components of the Nup107–160 complex affects progression through mitosis [30]. Taken together, LV-shRNA-mediated knock-down of Nup358/RanBP2, Nup214/CAN, Nup98 and Nup153 led to an efficient knock-down of the targeted nucleoporins and to minimal cytotoxic or cytostatic effects in the time frame of our experiments (up to 7 days p.t).

### Nucleoporin Depletion Disrupts Infectivity of HIV-1

**Figure 2:**
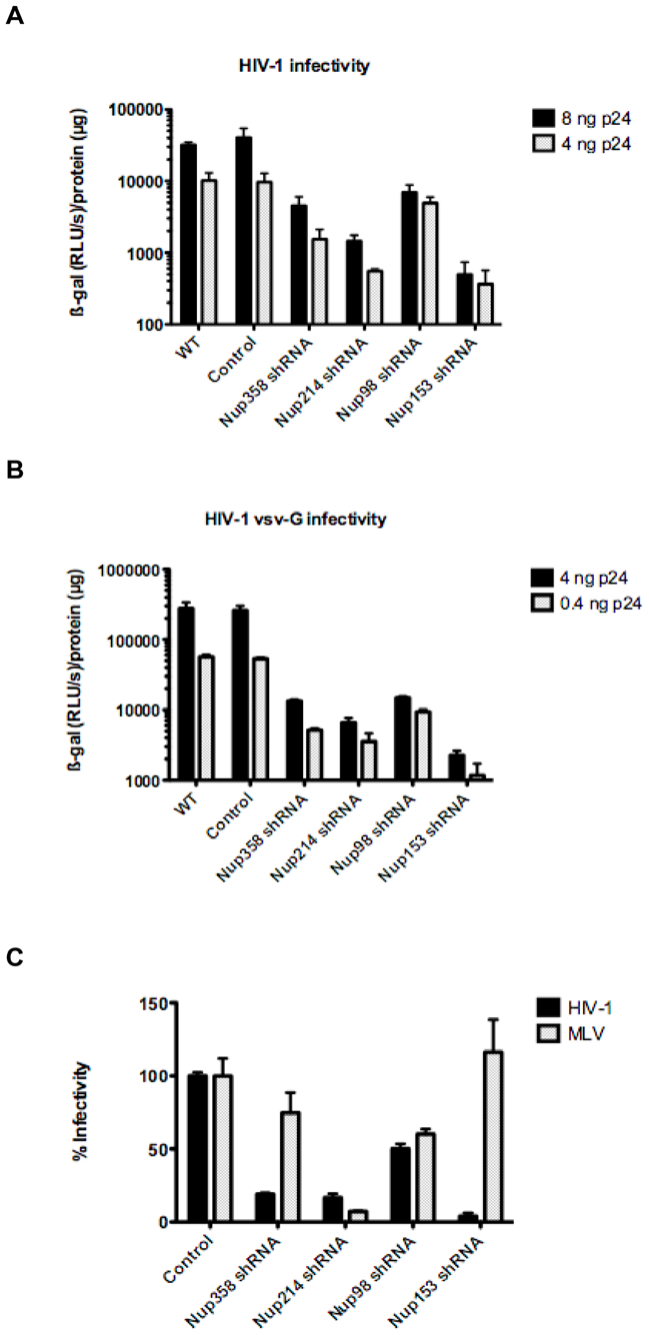
All tested nucleoporins disrupt HIV-1 infectivity when depleted but have only a limited effect on MLV infection. (**A**) The effect of nucleoporin knock-down on HIV-1 infectivity was assessed in P4-CCR5 indicator cells using a single cycle infectivity assay based on ß-galactosidase expression following infection with wild-type HIV-1 (8 or 4 ng) or (**B**) pseudotyped HIV-1 VSV-G (4 or 0.4 ng). Graphs show with log10 scale the mean luminescence values normalised for protein content +/− SEM and are each representative of 5 independent experiments. (**C**) Nup358/RanBP2, and Nup153 are specific for HIV-1 infection without effect on MLV infection. LV-shRNA transduced and control cells were infected with 5 ng of p24 of VSV-G pseudotyped NL4.3 luc virus and 5 ul of MLV luc/VSVG. Cell lysates were measured for luciferase activity 48 hr p.i. Luciferase values show averages of triplicate infections normalised by µg of proteins +/− SD.

We investigated the importance of the targeted nucleoporins on the early steps of HIV-1 infection in one-cycle infectivity assays. P4-CCR5 cells, which express ß-galactosidase under the control of the HIV-1 long terminal repeat (LTR) promoter transactivated by the viral tat protein, were infected at different MOI of wild-type and vesicular stomatitis virus G protein (VSV-G) pseudotyped HIV-1. Infectivity was measured 48 h post infection (p.i) and luminescence values were systematically normalised for live cell count using protein quantification. Depletion of all tested nucleoporins led to considerable reduction in HIV-1 infection, and with both viral envelopes thus ruling out possible variations due to the viral entry pathway (**Fig. 2A and 2B**). Infectivity defect ranged from 2-7-fold for Nup98 KD to over 2-log reduction for Nup153 KD in some experiments. Since knock-down of Nup98 leads to a concomitant knock-down of Nup96, which is derived from the same precursor [28], we verified the phenotype obtained with Nup98 KD cells using Nup98 knock-out (KO) mouse embryonic fibroblasts (MEFs), previously shown to specifically target Nup98 and not Nup96 [31]. Transduction of MEFs with TRIP-CMV-eGFP revealed a similar infectivity defect in specific Nup98 KO cells as that seen in Nup98 KD cells (∼4-fold, **Fig. S1A**), suggesting that the effects we observed with Nup98 knock-down cells are specific to depletion of Nup98. We conclude that depletion of all tested nucleoporins induces considerable disruption of infectivity as measured by single cycle HIV-1 infectivity assays. These results, importantly obtained in a stable knock-down system that offers greater versatility and longer testing conditions, are concordant with previous observations in transient KD cells [14], [17], [18], [15], [19], [20], [16], [32]. The efficiency of shRNA technology provided the opportunity to investigate in depth, and in robustly controlled and reproducible conditions, the involvement of each studied nucleoporin in experimentally separable steps of nuclear import (docking, translocation, integration).

Like other lentiviruses, HIV-1 has evolved an active import across the nuclear pore [12] which enables it to infect non-dividing cells. In contrast, the access of retroviruses such as the Murine Leukaemia Virus (MLV) to the host cell chromatin is dependent on mitosis and the associated disassembly of the nuclear envelope. To determine the specificity of each nucleoporin for HIV-1 infection, we investigated the effects of their depletion on MLV infection. P4-CCR5 cells were infected with either HIV-1-Luc or MLV-Luc at the same MOI and luciferase activity measured 48 h p.i. MLV infection was entirely unaffected by the depletion of Nup358/RanBP2, or Nup153 (**Fig. 2C**). Surprisingly however, knock-down of Nup214/CAN and Nup98 decreased both HIV-1 and MLV infectivity equally (**Fig. 2C**).

### Although All Studied Nucleoporins Perturb HIV-1 Infection when Depleted, Only Nup358/RanBP2, and Nup153 Affect its Nuclear Entry

**Figure 3:**
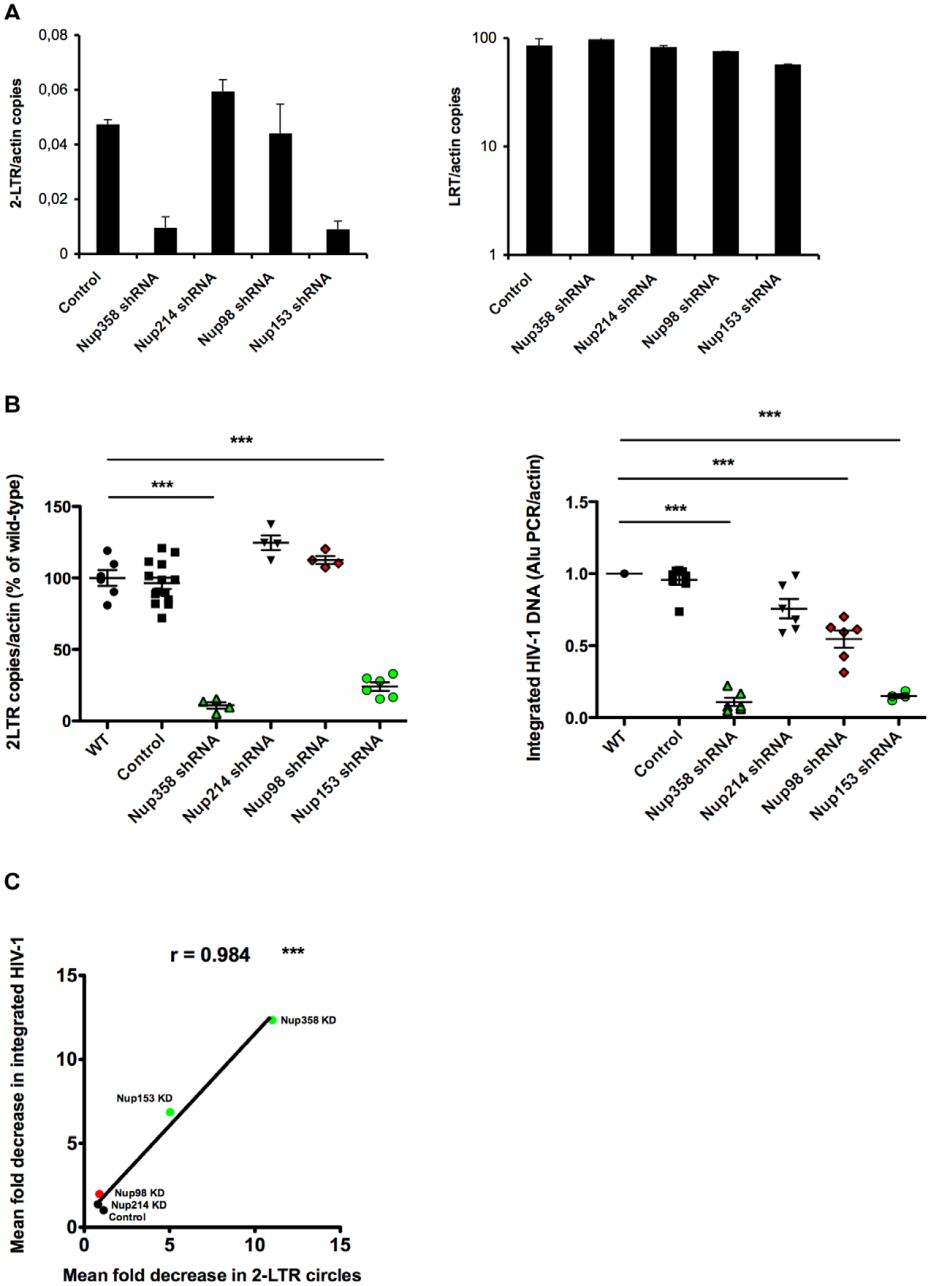
Implication of tested nucleoporins in HIV-1 nuclear import and integration. (**A**) P4-CCR5 cells depleted for each of the indicated nucleoporins and control cells were infected with VSV-G pseudotyped NL 4.3 luc virus. 2-LTR circle levels were assessed by quantitative PCR at 24 hr p.i (left panel). As a control for virus input, viral late reverse transcription (LRT) levels were analysed at 7 hr p.i (right panel, log10 scale). Both panels show average results of two independent infection experiments (mean ± SEM), normalised for actin copy number. Infections carried out in the presence of Nevirapine 5 µM led to undetectable levels of both 2-LTR circles and LRT. (**B**) HIV-1 nuclear import and integration were assessed in parallel in control and knock-down cells at 24 hr p.i. using quantitative PCR of 2-LTR circles and Alu-PCR, respectively. Graphs show individual values from three independent experiments with wild-type HIV-1 or HIV-1 VSV-G virus and the mean +/− SEM. Statistical significance was assessed using one-way Anova with Dunnet post-hoc (*** p<0.0001). (**C**) Correlation of mean fold decreases in 2-LTR circles and Alu-PCR signals obtained from panel B. Statistical significance was assessed by Pearson coefficient.

Having confirmed that all studied nucleoporins are involved in HIV-1 infection, we next sought to confirm the implication of these in HIV-1 nuclear import. Circular forms of non-integrated HIV-1 DNA containing two long terminal repeats (2-LTR) are found exclusively in the nucleus of infected cells and constitute convenient markers of nuclear import. We used quantitative PCR to measure 2-LTR circles in nucleoporin-depleted cells following infection [33]. Results revealed that only two out of the four tested nucleoporins, Nup358/RanBP2, and Nup153, led to a defect in HIV-1 nuclear import when depleted, while Nup214/CAN and Nup98, on the other hand, had no effect on HIV-1 nuclear import (**Fig. 3A**). No notable differences were observed between wild-type envelope and VSV-G pseudotyped HIV-1.

In addition to their involvement in nuclear import, nucleoporins have also been shown to participate in nuclear events such as chromatin remodelling and regulation of gene expression [22], [23]. In the case of HIV-1, recent studies suggest that nucleoporin knock-down can affect the efficiency of provirus integration and/or the selection of chromosomal sites for integration [18], [34]. We therefore tested the ability of HIV-1 to integrate within host chromatin in wild-type and knock-down cells using Alu-PCR in infected cells at 24 h p.i. Results showed a significant decrease in integrated HIV-1 DNA in cells depleted of Nup358/RanBP2, Nup98 and Nup153, but not Nup214/CAN (**Fig. 3B**). For most nucleoporins, this decrease was highly correlated to the decrease observed in 2-LTR circles (**Fig. 3B and 3C**), thus illustrating a general nuclear import defect rather than a specific block of integration following nuclear import. In the case of Nup98, however, depletion led to a 2-fold decrease in Alu-PCR signal compared to control, whereas no effect was observed for 2-LTR signals (**Fig. 3B and 3C**), suggesting that Nup98 might play a role in an HIV-1 integration step rather than translocation through the nuclear pore. This is discordant with a previous report suggesting a direct involvement of Nup98 in HIV-1 nuclear entry [14] but concordant with König *et al*. [18]. The observed defects in HIV-1 nuclear import and integration upon Nup153 depletion (<10-fold) are considerably more modest than the general defect in infectivity that we observed (>100-fold decrease). The depletion of Nup153 is associated with pleiotropic effects that likely account for this difference. Firstly, because of its involvement in nuclear basket integrity [35], Nup153 might be critical for the nucleocytoplasmic transport of host co-factors required for HIV-1 transcription or mRNA processing. Secondly, the potential involvement of Nup153 in the spatial organisation of chromatin [36] could mean that its depletion results in an overall decrease in transcription or in the integration of HIV-1 provirus in genomic sites unfavourable for transcription.

Several nucleoporins are known to be involved in mRNA export [37], [38], [39]. Since Nup214/CAN had no role in HIV-1 nuclear import or integration, we tested whether it might be involved in RNA export. We isolated and compared levels of cytoplasmic and nuclear RNA from control and knock-down cells. Cells depleted in Nup214/CAN and Nup153 had a 70% and 50% reduction, respectively, in cytoplasmic RNA relative to the nuclear fraction (**Fig. S1B**), confirming their role in a generic nuclear export [27], [40]. We conclude that, even though Nup214 was identified as a potential factor involved in HIV infection [18], the reduction on HIV-1 infectivity is in fact linked to a non-specific inhibition of RNA export, and consequently ß-galactosidase or luciferase mRNA export. The inhibition of RNA nuclear export accounts for the partial cytotoxicity measured in Nup214/CAN cells by MTT assay (**Fig. 1C**) and explains why both HIV-1 and MLV infections are equally affected by Nup214/CAN depletion (**Fig. 2C**).

### Nup358/RanBP2 Mediates HIV-1 Docking at the Nuclear Pore

**Figure 4:**
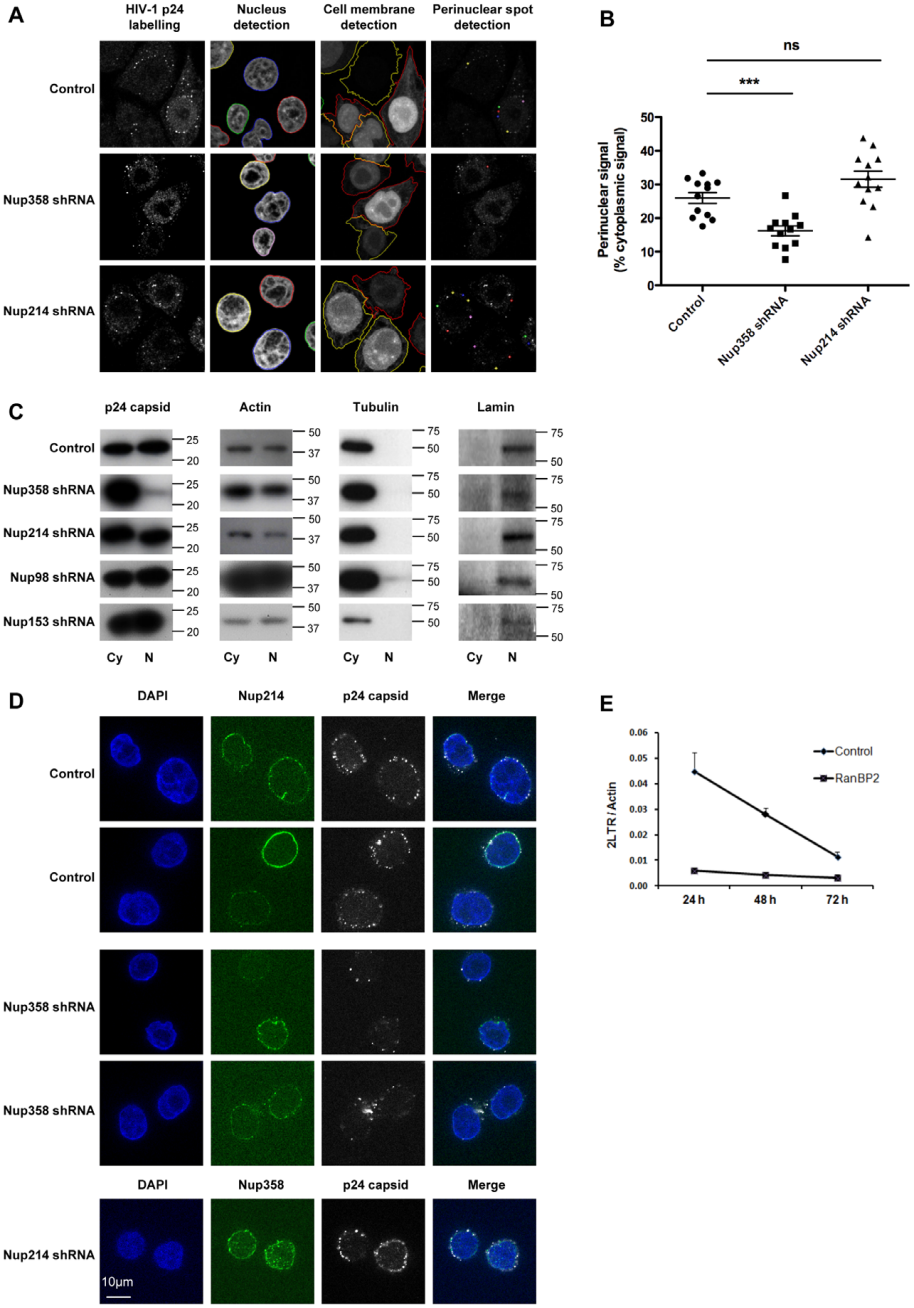
Nup358/RanBP2 mediates HIV-1 docking at the nuclear pore. (**A**) Control and Nup214/CAN or Nup358/RanBP2 knock-down cells were infected at 5 days p.t with HIV-1 VSV-G and fixed at 6 h p.i. Cells were labelled with anti-p24 HIV-1 capsid antibody, stained with Hoechst, and observed by confocal fluorescence microscopy. An Acapella script was used to segment the nucleus and cytoplasm of each cell. Nuclear and cell membrane detection were derived from Hoechst staining and LV-encoded GFP expression, respectively. HIV-1 p24 capsid signals were automatically quantified within perinuclear or cytoplasmic regions using a spot detection algorithm, with an intensity threshold set on negative controls. Perinuclear spots, defined as those present within 2 pixels of the nuclear membrane (480 nm), are highlighted as coloured dots in right-hand panels. (**B**) Quantification of Acapella-detected perinuclear p24 signal. Each point corresponds to one random cell and indicates the number of perinuclear capsid signals as a percentage of total cytoplasmic signal, representative of three independent experiments with mean +/− SEM. (**C**) Control or LV-shRNA transduced cells were infected with HIV-1 VSV-G and fractionated at 6 hr p.i. At this early time point, reverse transcription is not completed and the majority of viral complexes is still in the cytoplasm: p24 capsid signal in the nuclear fraction corresponds to viruses docked at the nuclear membrane. Nuclear and cytoplasmic fractions were tested for p24 content by Western blotting. Actin, tubulin and lamin labelling were carried out on the same samples to test for protein loading and integrity of cytoplasmic and nuclear fractions, respectively. Numbers indicate sizes in kDa. (**D**) Control and Nup358/RanBP2 knock-down cells were infected with HIV-1 VSV-G and fractionated at 6 hr p.i. Nuclei were labelled with anti-p24 and anti-Nup214 or -Nup358 antibodies, stained with Hoechst and placed on coverslips for microscopy observation using Apotome structured illumination (Zeiss). (**E**) 2-LTR time points in control and Nup358/RanBP2 knock-down cells at 24 h, 48 h and 72 h p.i normalised for actin copy number, mean of triplicates +/− SD.

At present, it is not known how HIV-1 docks at the nuclear pore, nor which nucleoporins if any are responsible for mediating this interaction. Since Nup358/RanBP2 and Nup214/CAN are both cytoplasmically oriented nucleoporins, we hypothesised that they might play a role in assisting HIV-1 docking at the NPC. Our previous work suggests that HIV-1 docks at the nuclear membrane as a complex still containing the viral capsid and that uncoating occurs at the nuclear pore upon completion of reverse transcription [41], [42]. Recent work also suggests that HIV-1 capsid may be important for mediating interactions with the nuclear transport machinery or the nuclear pore itself, and that capsid mutations can disrupt these interactions and HIV-1 nuclear import [43], [44], [19], [20]. At 6 h p.i, capsid (p24) fluorescent signal appears as a punctate pattern throughout the cytoplasm and at the nuclear envelope (**Fig. 4A**, left-hand panels). These bright spots are distinct from the weak and hazy p24 signal that may occasionally be observed in cell nuclei and that likely corresponds to primary antibody non-specific background signal. Our previous work supports that p24 signal at the nuclear rim corresponds to intact capsid cores [41]. A similar punctate pattern is observed with FlAsH-labelling of integrase at 6 h p.i. [45]. To assess whether Nup358/RanBP2 or Nup214/CAN mediate HIV-1 docking at the nuclear envelope, we measured the presence of p24 signal in LV-shRNA transduced and control cells at 6 h p.i. To obtain statistically robust comparison of control and knock-down cells, we used a spot detection software following the location of the nuclear and plasma membranes, and quantified individual p24 spots within 2 pixels of the nuclear membrane (480 nm, **Fig. 4A**). Around 100 cells per sample from 3 independent experiments were analysed. Using this approach, we found that ∼30% of all cytoplasmic p24 signal was perinuclear in control and Nup214/CAN knock-down cells at 6 h p.i (**Fig. 4B**). In contrast, depletion of Nup358/RanBP2 led to a decrease in perinuclear p24 signal (∼15% of total cytoplasmic signal), suggesting that Nup358/RanBP2 is involved in HIV-1 docking at the nuclear envelope. Although Nup358/RanBP2 has been shown to associate with microtubules for kinetochore assembly during mitotic nuclear envelope breakdown [26], [46], we do not think that this can account for the changes in HIV-1 localisation that we observe upon Nup358/RanBP2 depletion. Indeed, Nup358/RanBP2 does not play a role in cargo movement along microtubules and our microscopy observations were limited to interphasic cells when Nup358/RanBP2’s main location is at the cytoplasmic side of NPC.

To confirm these results using a biochemical assay, and to better distinguish between cytoplasmic and nuclear membrane-associated HIV-1 p24, we carried out cell fractionation in control and all knock-down cells at 6 h p.i, and tested for p24 content in cytoplasmic and nuclear fractions using Western blotting (**Fig. 4C**). At this time point, p24 signal detected within the nuclear fraction at 6 h p.i corresponds to viral complexes docked at the nuclear membrane and not to intranuclear p24 (**Fig. 4A**) [41], [42]. Successful fractionation was monitored using cytoplasmic and nuclear markers (tubulin and lamin A/C, respectively) in addition to actin loading control. Furthermore, we verified that the fractionation protocol does not perturb the nuclear rim localisation of Nup358/RanBP2 or Nup214/CAN using confocal fluorescence microscopy (**Fig. S2**). At 6 h p.i, nuclear membrane p24 signal was detected for control cells and cells depleted for Nup214/CAN, Nup98 and Nup153 (**Fig. 4C**). In knock-down cells for Nup358/RanBP2, in contrast, nuclear signal was distinctly low, indicating a defect in docking at the nuclear membrane. To confirm that p24 signal detected in the nuclear fraction is associated with the nuclear membrane and co-localises with NPCs, we labelled fractionated nuclei with appropriate antibodies and monitored protein localisation using microscopy. In control cells, p24 signal formed a ring of punctate labelling coincident with the Nup214 ring (**Fig. 4D**). In Nup214 knock-down cells, p24 signal was also found as a ring of fluorescent spots around the nucleus. In contrast, p24 signal in nuclear fractions of Nup358 depleted cells was strongly reduced, highlighting the ability of Nup358/RanBP2 to mediate intracellular HIV-1 docking at the nuclear membrane (**Fig. 4D**). Furthermore, a kinetic measurement of 2-LTR circles in Nup358/RanBP2 depleted cells compared to wild-type controls at 24 h, 48 h, and 72 h p.i. indicated that HIV-1 nuclear import is not delayed but blocked (**Fig. 4E**), suggesting that Nup358/RanBP2 is necessary for HIV-1 docking.

### Nup358/RanBP2 Interacts with HIV-1 *in vitro* Assembled CA-NC

**Figure 5:**
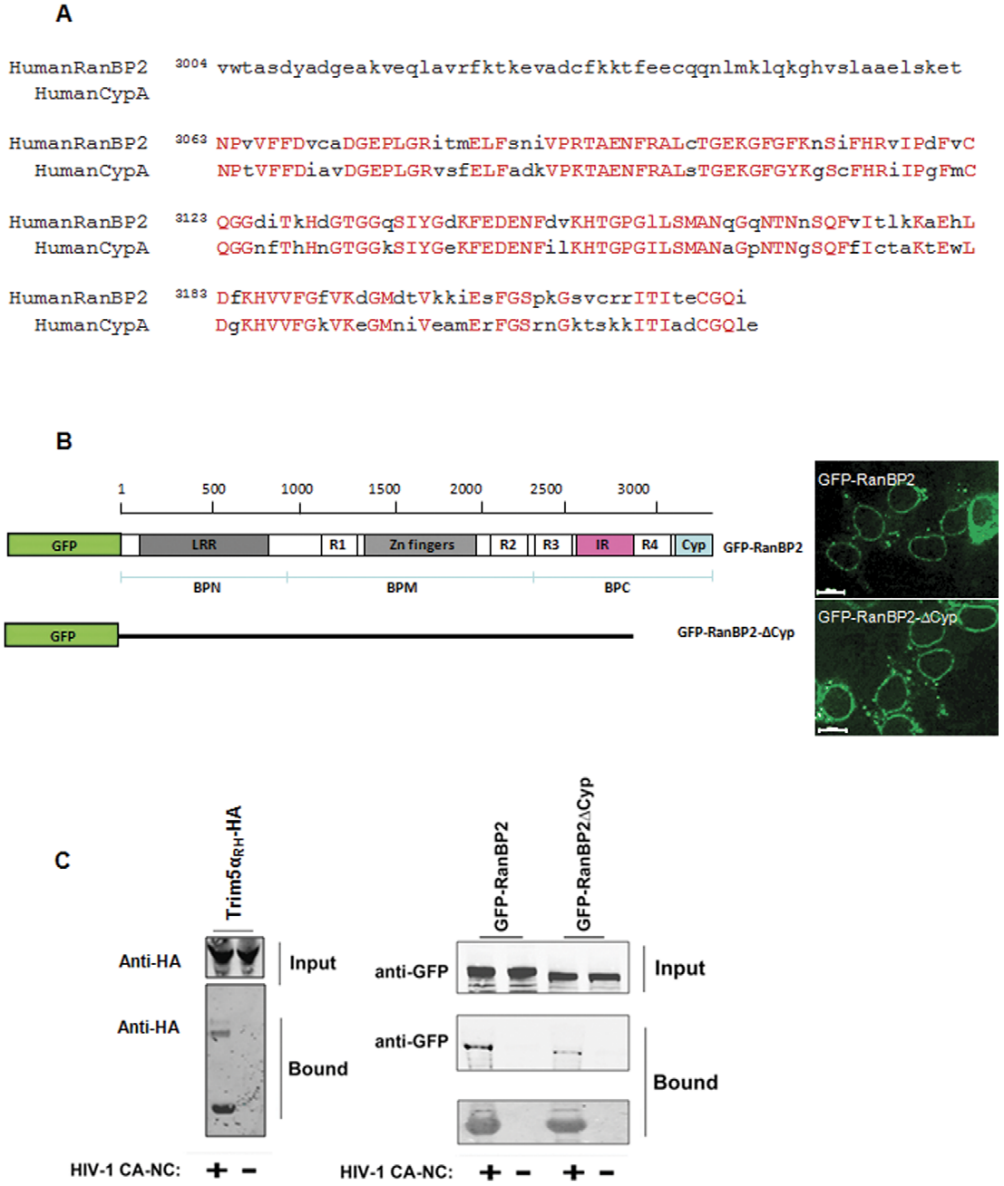
Nup358/RanBP2 interacts with HIV-1 CA-NC. (**A**) Protein sequence alignment of cyclophilin-homology domain of Nup358/RanBP2 and the human cyclophilin A, showing 66% identity (amino acids conserved are in red in capital letters and the amino acids divergent are in lowercase in black). (**B**) Domain structure of the wild-type and truncated Nup358-RanBP2 GFP fusion proteins used for interaction with CA-NC complexes. LRR: leucine-rich region. R1–R4: RanBP homology domains (RBH-1-4). Zn fingers: zinc finger domains. IR: internal repeats. Cyp: cyclophilin-homology domain. BPN, BPM, BPC: N-terminal, middle and C-terminal regions of Nup358/RanBP2 (Joseph and Dasso, 2008). Microscopy image showing location of the GFP-RanBP2 constructs in 293T transfected cells. (**C**) *In vitro*-assembled CA–NC complexes were mixed with 293T lysates containing WT GFP-RanBP2 or GFP-RanBP2-ΔCyp or Trim5α_RH_-HA and layered onto 70% sucrose before centrifugation. Immediately before mixing, an aliquot of the cell lysate was removed and blotted with α-GFP or α-HA antibodies to determine the steady-state expression levels of the different transgenic proteins (input). After centrifugation, the pellet was resuspended in SDS sample buffer and analyzed by Western blotting with an anti-GFP antibody (to detect RanBP2) or anti-HA antibody (to detect Trim5α_RH_) or an anti-p24 antibody (to detect CA-NC). As control of the assay we included TRIM5α-HA.

Based on these results, we next asked whether Nup358/RanBP2 interacts with HIV-1 cores. For this purpose, we tested the binding of Nup358/RanBP2 to *in vitro* assembled CA-NC complexes that recapitulate the architecture of the *bona fide* HIV-1 core [47] and were previously used to demonstrate the interaction between rhesus TRIM5α (TRIM5α_rh_) and the HIV-1 core [48]. In particular, Nup358/RanBP2 contains a cyclophilin homologous domain [49] that bears a high degree of homology with cyclophilin A (**Fig. 5A**), a well-established HIV-1 capsid interactant [50]. We therefore hypothesised that HIV-1 capsid docks at the nuclear pore via an interaction with the cyclophilin domain of Nup358/RanBP2. To test the implication of the RanBP2 CypA homology domain in interaction with capsid, we deleted the C-terminal residues 2787–3224 encompassing the cyclophilin-homology domain and RanBP homology region 4 (RBH4). We incubated 293T cell lysate expressing the full-length fusion protein GFP-RanBP2 or the GFP-RanBP2-ΔCyp deletion mutant with HIV-1 CA–NC complexes assembled *in vitro*, as previously described [47], [51]. We verified that fusion of Nup358/RanBP2 to GFP did not disrupt localisation at the nuclear membrane and that the folding of the C-terminal deletion mutant GFP-RanBP2- ΔCyp allowed correct localisation at the nuclear membrane (**Fig. 5B**).

After incubation of wild-type and mutant GFP-RanBP2 proteins with CA–NC complexes, samples were spun through a 70% sucrose cushion, and the pellets were analysed for the presence of Nup358/RanBP2 and HIV-1 p24 capsid. Results revealed that, like TRIM5α_RH_, Nup358/RanBP2 binds *in vitro* assembled HIV-1 CA-NC tubes that mimic the capsid lattice of mature viral cores (**Fig. 5C**). We observed a decreased binding of the deletion mutant lacking the C-terminus domain to HIV-1 CA-NC complexes when compared to wild-type. Fluorescent quantification of three independent binding experiments revealed that GFP-RanBP2-ΔCyp bound HIV-1 CA-NC complexes with decreased affinity. The ratio bound/input for GFP-RanBP2 and GFP-RanBP2-ΔCyp is 1±0.19 and 0.39±0.2, respectively (**Fig. 5C**). These experiments showed that GFP-RanBP2-ΔCyp binds ∼3-fold less than wild type in vitro assembled HIV-1 CA-NC complexes. These results suggested that the C-terminus region of Nup358/RanBP2 contributes to the ability of Nup358/RanBP2 to bind HIV-1 CA-NC, but that other parts of the protein, might also contribute to guaranty an efficient binding between the cytoplasmic filaments of the NPC, RanBP2 and HIV-1 CA, supporting the notion that Nup358/RanBP2 is involved in the docking for HIV at the nuclear pore.

## Discussion

Our study set out to identify the implication of nucleoporins, previously found to be involved in HIV-1 infectivity and/or nuclear import, in the actual translocation process through the NPC. We found that although all studied nucleoporins impaired HIV-1 infection upon depletion, only two (Nup358/RanBP2, and Nup153) were involved in nuclear import and indeed only one (Nup153) showed any evidence of actual participation in translocation through the NPC. This observation emphasises that the manipulation of NPCs by viruses is complex and not limited to mere translocation through the nuclear pore. Viruses may usurp cellular nucleoporins for docking [52], [53], [54], [55], [56], chromosomal site selection for integration [34], and disruption of nucleo-cytoplasmic trafficking [57], [58].

We previously hypothesised that HIV-1 capsids might dock directly on cytoplasmic filaments of the nuclear pore, based on the confined vibratory movement of HIV-1 complexes docked at the nuclear membrane [59], and the localisation of capsids at the nuclear pore frequently off-centered relative to the lumen of the pore [41]. Here we show that HIV-1 capsid cores bind to Nup358/RanBP2, thought to be the main component of NPC cytoplasmic filaments [7]. Interestingly, NPCs lacking Nup358/RanBP2 and devoid of cytoplasmic filaments were shown to maintain importin alpha/beta- and transportin-dependent import [7], which emphasises that depletion of Nup358/RanBP2 specifically impairs HIV-1 docking at the NPC rather than disrupting importin−/transportin-mediated nuclear import of HIV-1. Previous evidence suggests that Nup214/CAN mediates NPC docking of adenoviruses [52] and of herpes virus together with importin ß and Nup358/RanBP2 [54], [53], [55], [56]. In the case of HIV-1, data here show that Nup214/CAN is not involved in viral docking or nuclear import but that its apparent effect on HIV-1 infectivity is limited to a non-specific inhibition of mRNA export.

After entry in target cells, HIV-1 sheds its capsid shell during a step referred to as uncoating. However, where and when this occurs and the nature of the trigger (cellular or viral) remain a matter of debate. Reports of cytoplasmic HIV-1 complexes of broadly varying sizes and shapes suggest that uncoating could occur gradually during transport to the nuclear envelope [60], [42], presumably in response to successive changes in the cellular environment. However, increasing data supports the presence of HIV-1 capsid at the nuclear pore and/or acting as a determinant of HIV-1 nuclear import [61], [43], [41], [61], [44], [19], [20], [34]. Uncoating certainly also occurs during cytoplasmic transport, and possibly accounts for the majority of incoming viral complexes. However, these may correspond to viral complexes destined for or undergoing degradation, for instance following entry by endocytosis [62]. Our work shows that HIV-1 capsid interacts with Nup358/RanBP2 (**Fig. 5C**) and that depletion of Nup358/RanBP2 impairs arrival of HIV-1 complexes at the nuclear envelope, thus confirming the presence of HIV-1 capsid cores at the nuclear membrane (**Fig. 4**). However, our study does not show any effect for Nup358 in integration, since the strong reduction in proviral integration is simply due to a strong nuclear import defect (**Fig. 3B, C**) and considering the exclusive cytoplasmic location of Nup358/RanBP2 we do not expect to find its potential viral partner in the nucleus. We cannot exclude that the absence of this nucleoporin could affect HIV-1 site integration [34] but that may reflect the change of chromatin environment associated with depletion of Nup358 [26], [63], [46]. In this work, we identified Nup358/RanBP2 as docking factor for HIV-1 capsid using independent techniques to assess docking (microscopy, cell fractionation) and interaction (*in vitro* CA-NC binding). We are the first to identify the binding of Nup358/RanBP2 to HIV-1 *in vitro* assembled CA-NC complexes suggesting that this interaction is important for HIV-1 nuclear import.

Nup358/RanBP2 is one of over 20 human proteins that contain a cyclophilin-like domain [64]. The high homology between the human Nup358/RanBP2 cyclophilin-like domain and human CypA likely accounts for its ability to mediate docking of HIV-1 capsids at the nuclear pore. Indeed, a direct interaction between the Nup358/RanBP2 cyclophilin domain and HIV-1 CA N-terminal domain (NTD) was recently shown by calorimetric assay [32]. In this study, we demonstrate binding between in vitro assembled HIV-1 CA-NC and full-length Nup358/RanBP2, and show ∼3-fold reduced binding for GFP-RanBP2-ΔCyp deletion mutant when compared to GFP-RanBP2, suggesting the importance of this region that contains a cyclophilin-homology domain to have an efficient docking step of HIV at the NPC. Although we cannot exclude that a misfolding of the ΔCyp mutant accounts for reduced binding to HIV-1 cores, the mutant localised correctly at the nuclear membrane and did not form aggregates that precipitate in the absence of capsid, suggesting that this is an unlikely explanation. Interestingly, ΔCyp mutant maintained residual ability to interact with HIV-1 cores suggesting that other Nup358/RanBP2 domains, may also contribute to this binding. FG domains localised throughout the protein could represent the other part of the Nup358/RanBP2 with residual binding activity for HIV-1 CA-NC. Future experiments will be carried out to identify the amino acid residues of Nup358/RanBP2 essential for binding to HIV-1 cores.

In our study, Nup153, located in the nuclear basket of NPC, was the only nucleoporin involved in HIV-1 translocation through the nuclear pore, rather than in other NPC-related steps. Interestingly, it is the only nucleoporin that was identified in all four HIV genome-wide screens [17], [18], [65], [66]. We agree with other reports showing Nup153 involved in HIV-1 nuclear import [18], [15], [20]. The position of Nup153 in the nuclear basket leads us to hypothesise that exit from the nuclear basket, rather than entry into the lumen of the pore, might be critical for passage of HIV-1 into the nucleus. Based on recent single cargo translocation imaging [67], initial translocation of HIV-1 may involve a series of low-affinity, non-covalent and reversible interactions with FG domains from multiple Nups and diffusion through the NPC, whereas interaction with Nup153 might be critical for mediating irreversible and directional exit from the NPC. Interaction with FG-repeats may be mediated indirectly by nuclear transport receptors, such as importin 7 [68], [69], [70] or transportin 3 [17], [71].

After exit from the nuclear basket, both Nup98 and Nup153, which shuttle on and off the NPC [72] and have been shown to interact with chromatin [22], [23], [36], might accompany the PIC to its integration site. In particular, Nup98 has been found to localise to the nucleoplasm and participate in chromosomal remodelling and regulation of gene expression [22], [23], [72]. It will be interesting to determine whether Nup98 and/or Nup153 are hijacked by HIV-1 for transport to euchromatin and contribute to specific site selection in expressed genes, since previous work has shown that HIV-1 integrates preferentially within actively transcribed genes [73], [74]. Surprisingly, Nup98 depletion affected HIV-1 and MLV infection equally, but the reduction of MLV infectivity could merely be due to the slight accumulation of cells in G1/S phase that we observed since MLV enters the nucleus during metaphase.

**Figure 6:**
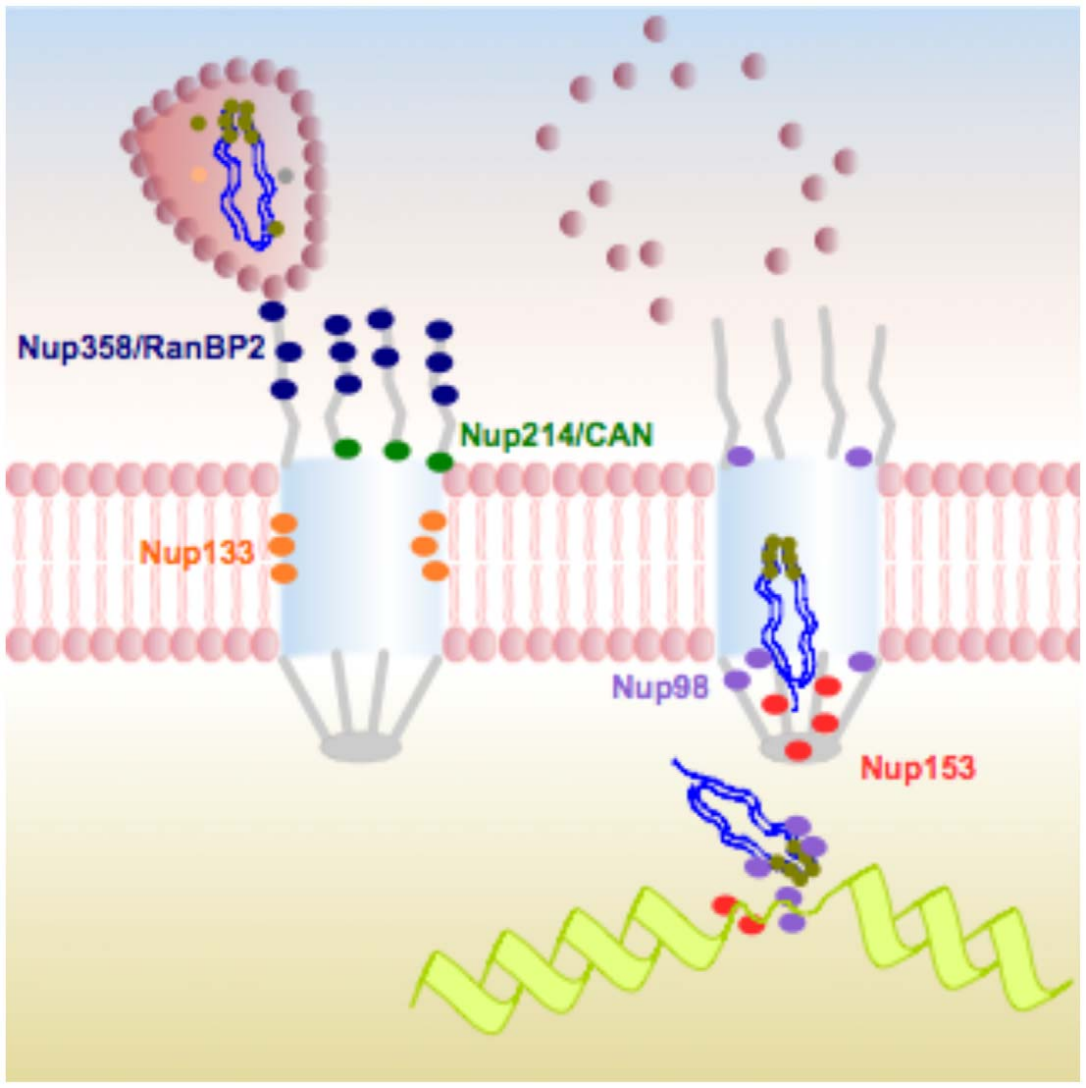
Overview of the functional interactions between nucleoporins and HIV-1 during infection. Left: HIV-1 capsid cores dock at the cytoplasmic filaments of NPCs via an interaction with Nup358/RanBP2. Nup214/CAN does not participate in HIV-1 docking or nuclear import. Its identification as a HIV-1 infectivity co-factor in previous studies is linked to its importance in RNA export from the nucleus. Right: upon uncoating, the viral pre-integration complex (PIC) crosses the lumen of the NPC and exits from the nuclear basket assisted by Nup153. Nup98 does not mediate HIV-1 nuclear import but has a moderate effect on HIV-1 integration in host chromatin.

Our work demonstrates that a key to the ability of HIV-1 to replicate in non-dividing cells is its capacity to use NPC components for its active transport across the nuclear pore, thus underlining the evolutionary adaptability of HIV-1 to exploit host mechanisms to achieve active nuclear import. Our study suggests a new appealing role for the NPC in HIV-1 infection proposing that the viral nuclear entry step may be important not only for actual translocation, but also for correct subsequent integration as a result of the physical interaction that exists between nuclear pore baskets and the chromatin (**Fig. 6**). The study of the physical and functional interactions between HIV-1 and the NPC not only contributes to our understanding of how other viruses manipulate the nuclear pore but also strengthen our comprehension of lentiviral vectors used for gene transfer protocols, whose active nuclear import is similar to that of HIV-1.

## Materials and Methods

### Cells and Viruses

The P4-CCR5 reporter cells are HeLa CD4+ CXCR4+ CCR5+ carrying the LacZ gene under the control of the HIV-1 long terminal repeat (LTR) promoter [75]. 293T cells are human embryonic kidney cells. The viral molecular clones used were based on LAI and called HIV-1 (wild-type), HIV-1-Luc, which contains the luciferase gene at the place of *Nef*, and LAIΔenv [76]. Viruses were produced by transient transfection of 293T cells using calcium phosphate precipitation with proviral plasmid alone or co-transfected with the Vesicular Stomatitis Virus glycoprotein (VSV-G) envelope expression plasmid pHCMV-G (Yee *et al*., 1994). Viruses were harvested at 48 hr post-transfection and treated with 25 U/mL of DnaseI (Roche) and 100 mM MgCl2 for 30 min at 37°C. Virus yield was measured by p24 ELISA according to the manufacturer’s instructions (Perkin Elmer). Retroviral vector, MLV luc, derived from Moloney was produced by co-transfection with calcium phosphate of pFBluc 10 µg, pCG gag-pol 10 µg, pMD2 VSV-G. The deletion mutant GFP-RanBP2-ΔCyp was generated by DNA restriction with KpnI of the GFP-RanBP2 plasmid.

### Lentiviral Vector-shRNA Construction and Production

LV-shRNA vectors are based on the TRIP-CMV-eGFP vector [77], which is ΔU3, contains the cis-acting sequences required for formation of the central DNA Flap, and encodes the enhanced green fluorescent protein (eGFP) under the control of the CMV promoter to monitor transduction. Complementary oligonucleotides coding for shRNAs (**Table S1**) were first annealed and cloned into *Bgl*II/*Hind*III of pSUPER (OligoEngine) downstream of the H1 promoter. The H1-shRNA cassette was then inserted into a polylinker within the U3 region of the vector plasmid. LV-shRNAs were produced by transient transfection of 293T cells with the vector, encapsidation (pCMVΔR 8.74), and VSV-G plasmids. Vectors were harvested 48 hr post-transfection and concentrated by ultracentrigation for 1 hr at 64,000 g (Beckman Coulter) at 4°C. LV-shRNAs were titered in P4-CCR5 cells using flow cytometry to assess GFP expression at 4 days post-transduction (p.t).

### Transductions and Infections

P4-CCR5 cells (4×10^6^) were transduced with LV-shRNA at MOI (multiplicity of infection) 25, 50 or 100 (1, 2, or 4×10^8^ transducing units (TU)) to generate stable knockdown cells referred to as 358KD, 214KD, 133KD, 98KD, and 153KD. Based on considerations of nucleoporin half-life and stability as well as cell viability, all knock-down cells were used at 5 days p.t except for 153KD (2 days p.t).

For ß-galactosidase and luciferase assays, 5,000 cells were infected in triplicate with HIV-1 (4 and 8 ng p24/well), HIV-1 VSV-G (0.4 and 4 ng), HIV-1 Luc (5 and 20 ng) or with 5 µl of MLV luc in 96-well plates. For quantitative PCR, 3×10^6^ cells were infected with HIV-1 env WT (2,000 ng p24 antigen) or HIV-1 VSV-G (500 ng p24 antigen) in T75 flasks for 2 hr at 37°C. All infection experiments were controlled by infection in the presence of 5 µM nevirapine. For docking experiments and confocal analysis we infected 200,000 cells with 100 ng of p24 antigen of HIV-1 VSV-G (MOI 50). For the biochemical fractionation 2×10^6^ cells were infected with 500 ng of p24 antigen of HIV-1 VSV-G (MOI 25).

### Western Blotting and Cellular Fractionation

Proteins were extracted on ice from WT and KD cells for 15 min using RIPA buffer, and protein concentration was quantified using the DC Protein Assay (Bio-Rad) with bovine serum albumin as standard. 100 µg of total protein lysate was loaded onto SDS-PAGE 6% Tris-glycine gel (Invitrogen) for Nup358/RanBP2, Nup214/CAN and Nup153, and 20 µg onto 4–12% Bis Tris gel (Invitrogen) for Nup98. Revelation was carried out using the ECL Plus Western Blotting kit (GE Healthcare).

For subcellular fractionation, 10^6^ cells were disrupted in 100mCSK buffer (10 mM Hepes pH 6.8, 10% (w/v) sucrose, 1 mM dithiothreitol, 1 mM MgCl_2_, EDTA-free protease inhibitor mixture (Roche Molecular Biochemicals), 100 mM NaCl and 0.5% Nonidet P-40) at 6 hr post-infection (p.i) to separate cytoplasmic and nuclear fractions as previously described [78]. The cells were lysed for 10 min on ice, and the nuclei were pelleted and washed with 100mCSK without NP-40. To extract the nuclear fraction, containing NPCs and lamin, nuclei were resuspended in 400mCSK buffer (same as 100mCSK, but containing 400 mM NaCl) and left on ice for 5 min followed by centrifugation at 7,500 rpm for 2 min. The same quantitative fraction of cytoplasm and nucleus was loaded into 4–12% Bis Tris gel (Invitrogen).

### Antibodies

Primary antibodies used for Western blotting (WB) and immunofluorescence (IF) were rabbit anti-RanBP2 (Affinity BioReagents, WB 1∶1,000, IF 1∶100), rabbit anti-Nup214 (Abcam, WB 1∶500, IF 1∶500), rat anti-Nup98 (Abcam, WB 1∶4,000, IF 1∶100), anti-mouse Nup153 (gift from B. Burke, WB 1∶500, IF 1∶10), mouse anti-p24 antibody (NIH 183-H12-5C), mouse anti-lamin A/C (Santa Cruz), mouse anti-alpha-tubulin (Sigma), and goat anti-RanGAP1 (Santa Cruz, IF 1∶500). WB secondary or conjugated antibodies were Beta Actin HRP conjugated antibody (Abcam, 1∶2,500), anti-mouse IgG HRP (GE Healthcare, 1∶5,000), anti-rabbit IgG HRP (GE Healthcare, 1∶5000), anti-rat IgG HRP (Sigma, 1∶80,000). IF secondary antibodies were goat anti-mouse Cy3 (Amersham, 1∶1000), goat anti-rabbit Alexa 488 (Invitrogen 1∶1000), goat anti-rat Alexa 488 (Southern Biotechnology, 1∶1000), donkey anti-goat Alexa 546 (Invitrogen 1∶1000), monoclonal antibodies directed against the HA epitope tags (Roche), rabbit anti-GFP (Clontech).

### HIV-1 CA-NC Expression and Purification

The HIV-1 CA-NC protein was expressed, purified and assembled as previously described [47]. The pET11a expression vector (Novagen) expressing the CA-NC protein of HIV-1 was used to transform BL-21(DE3) E. coli. CA-NC expression was induced with 1 mM isopropyl-β-D-thiogalactopyranoside (IPTG) when the culture reached an optical density of 0.6 at 600 nm. After 4 hours of induction, the cells were harvested and resuspended in 20 mM Tris-HCl (pH 7.5), 1 µM ZnCl_2_, 10 mM 2-mercaptoethanol and protease inhibitors (Roche). Lysis was performed by sonication, and debris were pelleted for 30 minutes at 35,000×g. Nucleic acids were stripped from the solution by using 0.11 equivalents of 2M (NH_4_)_2_SO_4_ and the same volume of 10% polyethylenimine. Nucleic acids were removed by stirring and centrifugation at 29,500×g for 15 minutes. The protein was recovered by addition of 0.35 equivalents of saturated (NH_4_)_2_SO_4_. The protein was centrifuged at 9,820×g for 15 minutes and resuspended in 100 mM NaCl, 20 mM Tris-HCl (pH 7.5), 1 µM ZnCl_2_ and 10 mM 2-mercaptoethanol. Lastly the CA-NC protein was dialyzed against 50 mM NaCl, 20 mM Tris-HCl (pH 7.5), 1 µM ZnCl_2_ and 10 mM 2-mercaptoethanol, and stored at -80°C.

### In Vitro Assembly of CA-NC Complexes

HIV-1 CA-NC particles were assembled in vitro by diluting the CA-NC protein to a concentration of 0.3 mM in 50 mM Tris-HCl (pH 8.0), 0.5 M NaCl and 2 mg/ml DNA oligo-(TG)50. The mixture was incubated at 4°C overnight and centrifuged at 8,600×g for 5 minutes. The pellet was resuspended in assembly buffer (50 mM Tris-HCl (pH 8.0), 0.5 M NaCl) at a final protein concentration of 0.15 mM [47], [79], and stored at 4°C until needed.

### Binding of GFP-RanBP2 to HIV-1 CA-NC Complexes

293T cells were transfected with plasmids expressing wild-type or mutant GFP-fusion RanBP2 proteins or Trim5α_RH_-HA. Forty-eight hours after transfection, cell lysates were prepared as follows: washed cells were resuspended in capsid-binding buffer (10 mM Tris, pH 7.4, 1.5 mM MgCl2, 10 mM KCl, 0.5 mM DTT). The cell suspension was frozen and thawed, and incubated on ice for 10 minutes. Next, the lysate was centrifuged in a refrigerated Eppendorf microcentrifuge (∼14,000×*g*) for 5 minutes. The supernatant was supplemented with 1/10 volume of 10X PBS and then used in the binding assay. To test binding, 5 µl of CA-NC particles assembled *in vitro* were incubated with 200 µl of cell lysate at room temperature for 1 hour. A portion of this mixture, henceforth referred to as “INPUT” was stored. The mixture was spun through a 70% sucrose cushion (70% sucrose, 1X PBS and 0.5 mM DTT) at 100,000×*g* in an SW55 rotor (Beckman) for 1 hour at 4°C. After centrifugation, the supernatant was carefully removed and the pellet was resuspended in 1X SDS-PAGE loading buffer and henceforth referred to as “BOUND”. The level of GFP-fusion RANBP2 proteins was determined by Western blotting with an anti-GFP antibody as described above. The level of HIV-1 CA-NC protein in the pellet was assessed by Western blotting with an anti-p24 capsid antibody.

### Microscopy Sample Preparation

Cells were seeded onto 12 mm diameter coverslips (Marienfeld) in 24-well plates the day before fixation or infection. Cells were fixed in 2% paraformaldehyde (Electron Microscopy Sciences) for 10 min, treated with 50 nM NH_4_Cl for 10 min, permeabilised with 0.5% triton for 15 min and blocked with 0.3% bovine serum albumin (BSA). All incubations were carried out at room temperature and were followed by 2–5 PBS washes. Cells were incubated with primary antibodies for 1 hr and secondary antibodies for 30 min. Antibodies were diluted in 0.3% BSA. Nuclei were stained with Hoechst (Invitrogen). Finally, cells were mounted onto glass slides (Thermo Scientific) with Vectashield (Vector Laboratories). The same protocol was used for nuclei isolated following subcellular fractionation with the difference that labelling was carried out in Eppendorf tubes.

### Imaging and Data Analysis

Confocal microscopy was carried out using a Leica SP5 confocal microscope with a 63x objective, using identical laser and exposure times for all samples. Images were acquired as 512×512 images and with line averaging of 3. For docking experiments, control and knock-down cells were fixed 6 hr p.i and labelled with mouse p24 antibody and anti-mouse Cy3 antibody. Nuclei were stained by Hoechst (Invitrogen). Confocal slices of 1 µm, and in the volume of the nucleus only, were acquired to avoid the detection of the same viruses in more than one image and to correctly distinguish perinuclear signal from cytoplasmic signal. To avoid any bias, sample preparation, image acquisition, and image analysis were done by 3 independent experimenters.

Image analysis was carried out using the Acapella software (Perkin Elmer). For docking experiments, an Acapella script was developed to segment the nucleus and cytoplasm of each cell. Capsid signals were automatically quantified within perinuclear or cytoplasmic regions using a spot detection algorithm (script available upon request). A parameter set was performed using negative controls (non-infected cells labelled with primary and secondary antibodies) and used to set an intensity threshold of 120 for spot detection. The same was then applied for control and knock-down sets of data. Flow cytometry data was analysed using FlowJo 6.4.1. Statistical analyses (unpaired t test and linear regressions) were performed using Prism 5.

### ß-galactosidase and Luciferase Assays

ß-galactosidase (Roche) and luciferase (Promega) activity was measured 48 hr p.i according to manufacturer’s instructions, using a microplate ﬂuorimeter (Victor, Perkin Elmer). Protein quantification by Bio-Rad protein assay was carried out on the same lysates to normalize the ß-galactosidase and luciferase data for protein content.

### MTT Assay

This assay tests cell viability based on the conversion of yellow MTT (3-(4,5-dimethylthiazole-2-yl)-2,5-diphenyl tetrazolium bromide) salt into blue formazan crystals through mitochondrial dehydrogenases. Cells were seeded in 96-well plates 24 hr prior to the assay (10,000 cells/well). 10 µl of MTT solution (Sigma, 50 mg/ml) were added to each well and incubated at 37°C for 3–4 hr. After medium removal, formazan crystals were solubilised with 100 µl of organic solvent (ethanol/DMSO 1∶1). Absorbance was measured by plate luminometry at 550–570 nm (Victor, Perkin Elmer).

### Propidium Iodide Labelling and Flow Cytometry

Cells were fixed in cold 1∶1 ethanol (80%)/Acetone for 1 hr at −20°C. After washing, cells resuspended in propidium iodide (10 µg/mL) and RNase (50 µg/ml) for 30 min at 37°C and analysed directly by flow cytometry.

### Quantitative PCR

Infected cells were treated for 30 min at 37°C with 1000 U of DnaseI (Roche) and total cellular DNA was then isolated using the QIAamp DNA micro kit (QIAGEN). The primer sets used to detect each sequence were as follows: DNA synthesis was analysed at 7 hr p.i by late RT forward, MH531∶5′-TGTGTGCCCGTCTGTTGTGT-3′; late RT reverse, MH532∶5′-GAGTCCTGCGTCGAGAGAGC-3′; late RT probe, LRT-P: 5′-(FAM)-CAGTGGCGCCCGAACAGGGA-(TAMRA)-3′. Two long terminal repeat (2-LTR) containing circles were detected using primers MH535/536 and probe MH603 [33], using as standard curve the pUC2LTR plasmid, which contains the HIV-1 2-LTR junction.

Assessment of integration by Alu-PCR was performed as previously described [80].

To rule out amplification of LV-shRNA, the 5′ primer used for the first amplification step was designed to recognise a unique sequence in HIV-1 U3 not present in LV-shRNA. As in Brussel and Sonigo, [80], this primer was extended with the lambda phage-specific heel sequence shown in bold (5′-**atgccacgtaagcgaaac**tttccgctggggactttccaggg). The U3 modified primer was used in combination with two Alu primers (5′-tcccagctactggggaggctgagg) and (5′-gcctcccaaagtgctgggattacag). DNA generated from WT-infected cells was endpoint diluted in DNA prepared from uninfected cells to generate the integration standard curve and serial dilutions were made starting from 50,000 infected cells. Each sample amplified contained 10^4^ infected cells mixed at 40,000 uninfected cells. The control of the first round PCR was the amplification without Alu primers using rTth DNA polymerase XL as recommended by the manufacturer (Applied Biosystems Inc, Foster City, CA N808-0187). Dilution 1∶10 of the first round was amplified using the phage lambda Spa primer (5′-atgccacgtaagcgaaact**)** and U5-specific primer (5′- ctgactaaaagggtctgagg) with the probe (6FAM- ttaagcctcaataaagcttgccttgagtgc-TAMRA).

Both 2-LTR and Alu-PCR reactions were carried out at 24 hr p.i unless otherwise stated and normalised by amplification of the housekeeping gene β-Actin using the following primers and probe (5′-aacaccccagccatgtacgt), (5′-cggtgaggatcttcatgaggtagt), (6FAM-ccagccaggtccagacgcagga-BBQ). Reactions contained 1X FastStart Universal Probe Master Mix (Rox) 2X (Roche), 300 nM forward primer, 300 nM reverse primer, 100 nM probe primer and template DNA in a 20 µl volume. After initial annealing (50°C for 2 min) and denaturation steps (95°C for 15 min), 40 cycles of amplification were carried out (95°C for 15 s, 58°C for 30sec, and 72°C for 30 sec). As a control, single cycle ß-gal infectivity assays were systematically carried out to certify the phenotype. As control of the qPCR we treated all samples during the infection with Nevirapine 5 µM.

### Online Supplemental Material

#### RNA export assay

Nuclear and cytoplasmic RNA was extracted from control and knock-down cells using the PARIS kit (Ambion) according the manufacturer’s instructions. Cytoplasmic and nuclear RNA content was quantified with a Nanodrop ND-1000.

#### MEFs infection

Wild-type and Nup98KO mouse embryonic fibroblasts (MEFs) were transduced with the HIV-1-derived vector TRIP-CMV-eGFP (1,000, 100, 10 and 1 ng p24 per 0.3×10^6^ cells) and efficiency of transduction was measured 48 hr p.t using flow cytometry in duplicate.

## Supporting Information

Figure S1 **(A)** Effect of Nup98 knock-out on HIV-1 transduction efficiency. Wild-type and Nup98KO MEFs were transduced with TRIP-CMV-eGFP. GFP mean fluorescent intensity (MFI) at 48 hr p.t is plotted against vector dose for a representative experiment. **(B)** RNA export assay. Graph shows the mean ratio of cytoplasmic/nuclear RNA +/− SD, representative of 2 independent experiments.
(TIF)

Figure S2 **Following subcellular fractionation, nuclear fractions maintain a nuclear rim of cytoplasmic nucleoporins (Nup214/CAN and Nup358/RanBP2) indicating that the NPCs are not removed from the nuclear envelope.** Hela cells were kept either intact or treated with mild NP-40 to extract nuclei. Samples were fixed, permeabilised using 0.5% Triton X100 in the case of intact cells, labelled with anti-RanBP2 or -Nup214 specific antibodies, and deposited onto poly-lysine coating coverslips. Images were acquired using an Apotome structured illumination microscope (Zeiss).
(TIF)

Table S1 **Oligonucleotide sequences used for cloning of shRNAs.** siRNA sequences appear in bold.
(DOC)
