# Correction: Human Nucleoporins Promote HIV-1 Docking at the Nuclear Pore, Nuclear Import and Integration

**DOI:** 10.1371/annotation/34f46796-5d53-4a23-b3df-b599b0369eb9

**Published:** 2013-12-19

**Authors:** Francesca Di Nunzio, Anne Danckaert, Thomas Fricke, Patricio Perez, Juliette Fernandez, Emmanuelle Perret, Pascal Roux, Spencer Shorte, Pierre Charneau, Felipe Diaz-Griffero, Nathalie J. Arhel

There was an omission in the funding statement. Thomas Fricke (TF) was also supported by National Institutes of Health grant R01 AI087390.

There was also an error in Author Contributions. All authors participated in the writing of the manuscript. 

