# Supplementary figures and images for: Human Nucleoporins Promote HIV-1 Docking at the Nuclear Pore, Nuclear Import and Integration
Source: PLoS One. 2012 Sep 25;7(9):e46037. doi: 10.1371/journal.pone.0046037 (PMC3457934; doi:10.1371/journal.pone.0046037)

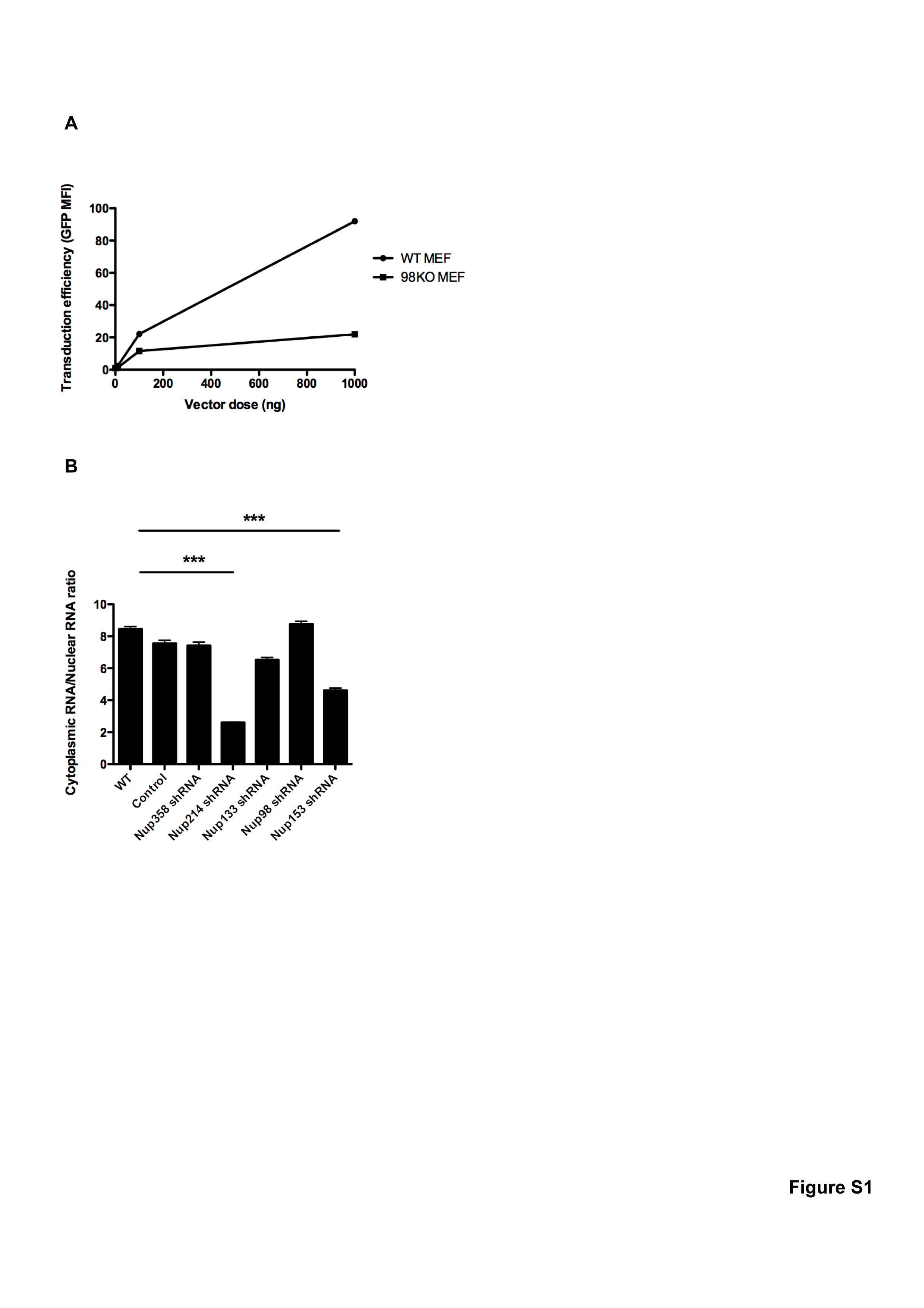

Supplement: Figure S1 — (A) Effect of Nup98 knock-out on HIV-1 transduction efficiency. Wild-type and Nup98KO MEFs were transduced with TRIP-CMV-eGFP. GFP mean fluorescent intensity (MFI) at 48 hr p.t is plotted against vector dose for a representative experiment. (B) RNA export assay. Graph shows the mean ratio of cytoplasmic/nuclear RNA +/− SD, representative of 2 independent experiments. (TIF) [file pone.0046037.s001.tif]

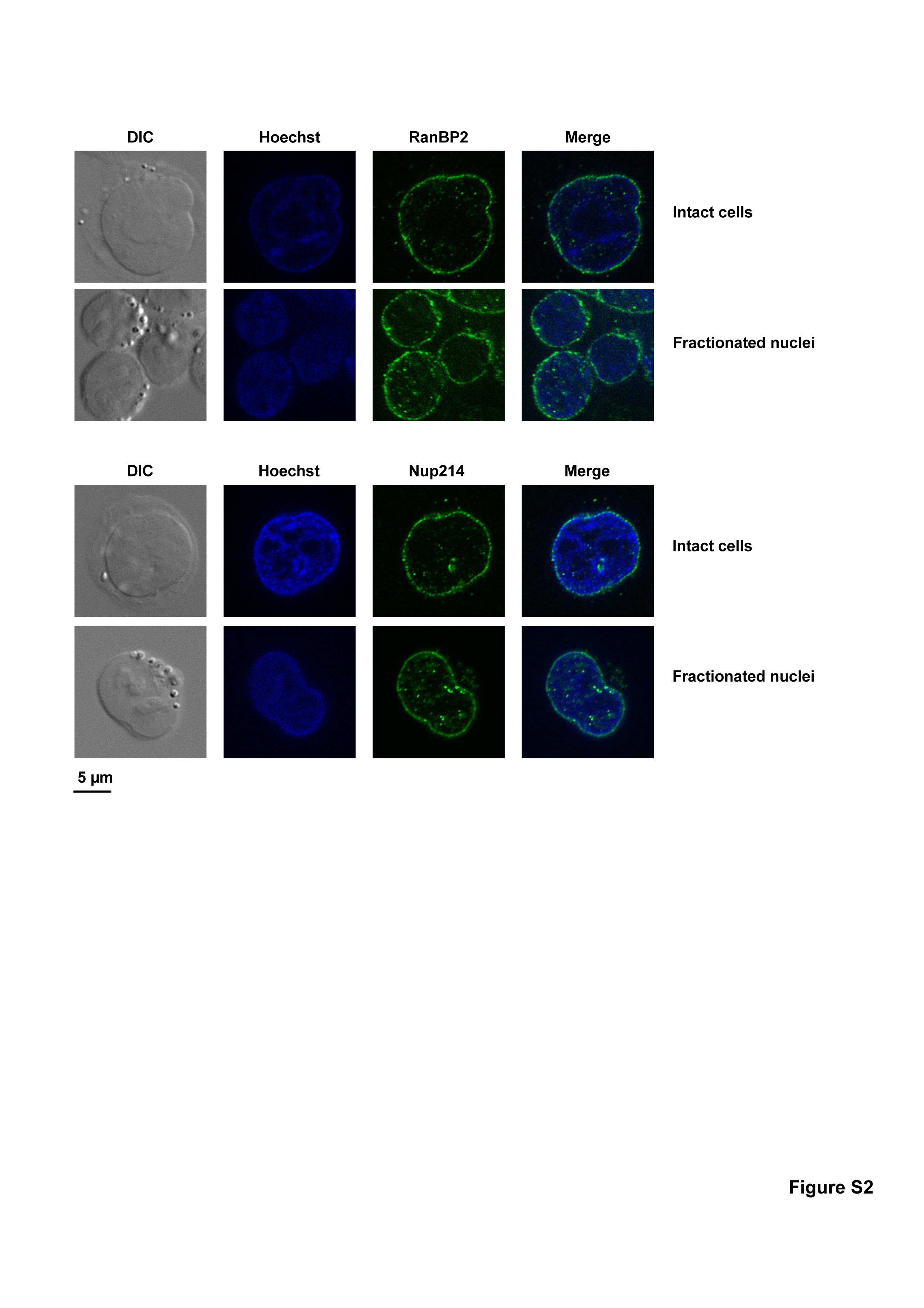

Supplement: Figure S2 — Following subcellular fractionation, nuclear fractions maintain a nuclear rim of cytoplasmic nucleoporins (Nup214/CAN and Nup358/RanBP2) indicating that the NPCs are not removed from the nuclear envelope. Hela cells were kept either intact or treated with mild NP-40 to extract nuclei. Samples were fixed, permeabilised using 0.5% Triton X100 in the case of intact cells, labelled with anti-RanBP2 or -Nup214 specific antibodies, and deposited onto poly-lysine coating coverslips. Images were acquired using an Apotome structured illumination microscope (Zeiss). (TIF) [file pone.0046037.s002.tif]
