# Supplementary material for: Human Nucleoporins Promote HIV-1 Docking at the Nuclear Pore, Nuclear Import and Integration
Source: PLoS One. 2012 Sep 25;7(9):e46037. doi: 10.1371/journal.pone.0046037 (PMC3457934; doi:10.1371/journal.pone.0046037)
Supplement: Table S1 — Oligonucleotide sequences used for cloning of shRNAs. siRNA sequences appear in bold. (DOC) [file pone.0046037.s003.doc]

| **Name** | **shRNA sequence** | **Reference** |
| --- | --- | --- |
| Nup358 | 5’-gatcccc**cacagacaaagccgttgaa**ttcaagaga**ttcaacggctttgtctgtg**ttttta  5’-agcttaaaaa**cacagacaaagccgttgaa**tctcttgaa**ttcaacggctttgtctgtg**ggg | Hutten and Kehlenbach, 2006 |
| Nup214 | 5’-gatcccc**gtcacggaaacagtgaaag**ttcaagaga**ctttcactgtttccgtgac**ttttta  5’**-**agcttaaaaa**gtcacggaaacagtgaaag**tctcttgaa**ctttcactgtttccgtgac**ggg | Hutten and Kehlenbach, 2006 |
| Nup98 | 5’-gatcccc**cagtgtattactgctatgaaa**ttcaagaga**tttcatagcagtaatacactg**ttttta  5’-agcttaaaaa**cagtgtattactgctatgaaa**tctcttgaa**tttcatagcagtaatacact**gggg | König et al., 2008 |
| Nup153 | 5’-gatcccc**agtgttcagtatgctgtgtttct**ttcaagaga**agaaacacagcatactgaacact**ttttta  5’-agcttaaaaa**agtgttcagtatgctgtgtttct**tctcttgaa**agaaacacagcatactgaacact**ggg | Mackay et al., 2009 |

**Table S3**
